# Enhanced Catalytic Probe Design for Mapping Radical Density in the Plasma Afterglow

**DOI:** 10.1021/acs.jpca.4c06195

**Published:** 2024-11-11

**Authors:** Anja Herrmann, Patrick M. Krebaum, Susanta Bera, Mihalis N. Tsampas, Mauritius C. M. van de Sanden

**Affiliations:** † Dutch Institute for Fundamental Energy Research (DIFFER), Eindhoven 5600 MB, Eindhoven, The Netherlands; ‡ Eindhoven Institute for Renewable Energy Systems (EIRES), Eindhoven University of Technology, Eindhoven 5600 MB, The Netherlands

## Abstract

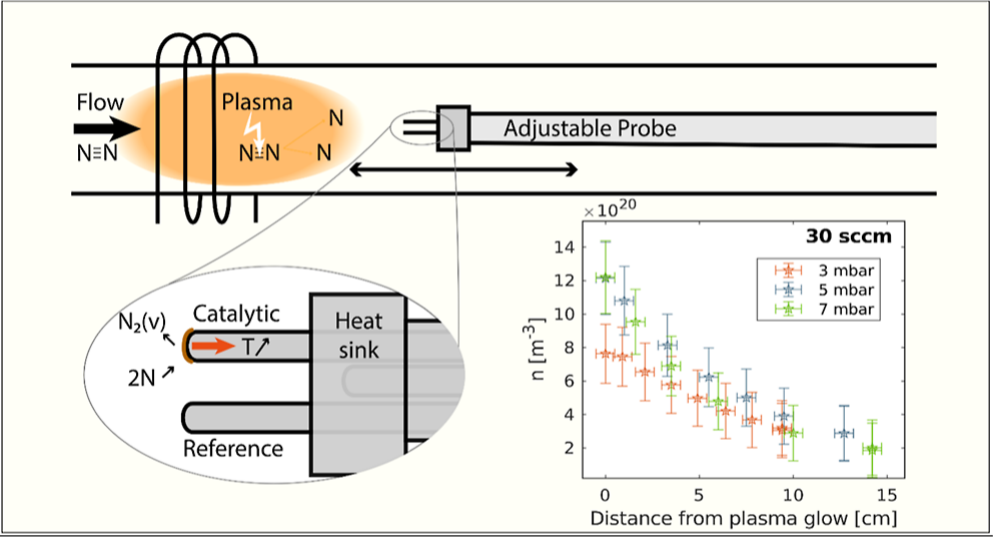

The electrification of chemical processes using plasma generates an increasing demand for sensors, monitoring concentrations of plasma-activated species such as radicals. Radical probes are a low-cost in situ method for spatially resolved quantification of the radical density in a plasma afterglow using the heat from the exothermal recombination of radicals on a catalytic surface. However, distinguishing recombination heating from other heat fluxes in the system is challenging. In this study, we present a heat flux analysis based on probe measurements inside the reactor, with simultaneous IR imaging monitoring of the temperature of the reactor wall. The impact of radiation heat on a single thermocouple as well as the advantage of a dual thermocouple setup (using a catalytic unit together with a reference thermocouple) is shown. We add a heat sink with a monitored temperature to the dual thermocouple setup, allowing the determination of conductive and radiative heat fluxes. The heat sink gives more information on the measurement and reduces ambiguities in the evaluation used by others. The probe was tested by mapping N atom densities throughout the plasma afterglow of our reactor, enabling evaluation of the recombination kinetics of the radicals in the gas phase. Three-body recombination was shown to be the main pathway of recombination, with a recombination rate of *k*_rec_ = (2.0 ± 0.9)·10^–44^ m^6^/s, which is in line with the known literature findings, demonstrating that the measured species are N radicals and the probe did not influence the plasma or recombination reactions in the afterglow.

## Introduction

The usage of plasma can be a powerful tool in power-to-X transformation, transforming electricity from renewable sources to chemical energy. Ignition of plasma involves applying an external electric field to gas, causing electrons to accelerate and transfer their newly gained energy to the gas molecules through collisions, creating highly reactive species. Examples of applications are energy storage through the production of synthetic fuels, CO_2_ capture, and the electrification of chemical processes such as nitrogen fixation.^1−4^ The common feature of all applications is the need for sensors capable of in situ measurement of the concentration of active species for process control and enhancement.

One of the activated species are radicals. These are atoms or molecules formed through dissociation of (larger) molecules. They are highly reactive due to having free valence electrons. A low-cost, in situ method for measuring radical flux densities in the afterglow of plasma uses radical probes.

Radical probes are based on a calorimetric principle: they measure the heat produced from the recombination of radicals on a surface with a known recombination probability. This principle was used as early as 1924 by Bonhoeffer who coated the bulb of a thermometer with various catalysts to study hydrogen radical recombination.^5^ The heat produced at the surface due to the recombination reactions is related to the number of atoms recombining, as well as the energy released per recombination reaction, which is related to the dissociation energy of the molecule (*W*_D_). The power of heating from recombination on the catalytic surface is calculated according to^6^
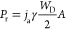
1with the recombination coefficient of atoms on the surface γ, the surface area *A* of the catalyst, and the atomic flux to the surface  containing the atomic density *n* and the thermal velocity *v*. If the recombination at the probe surface is too high, it can deplete atoms close to the surface, leading to a lower atomic flux. The amount of flux lost at a surface can be calculated according to^7^, with β being the surface reaction probability consisting of sticking and recombination coefficient. The size of the probe also plays a role in the total depletion of atoms, so it is generally suggested to keep the probes small.^8,9^ In case of no depletion, the atomic flux at the catalyst can then be calculated according to
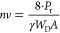
2

While probes using heat flux sensors have been proposed,^10,11^ sensors measuring the temperature of a catalyst over time are more commonly used. The main designs are thermocouple catalytic probes^9,12−16^ and fiber optical catalytic probes.^6,17,18^ Initially, the thermocouple catalytic probes were used for studying the recombination coefficients of surfaces in contact with radicals.^19,20^ Later, they were further developed for the detection of atomic density.^9,12−16,21^ The development of fiber optical catalytic probes, where the temperature of the catalyst is measured optically from its emitted heat, was introduced by Babič et al.^17^ All systems have in common that heating from recombination reactions must be distinguished from other heat fluxes in the system to gain a correct value for the radical flux.

**Figure 1:**
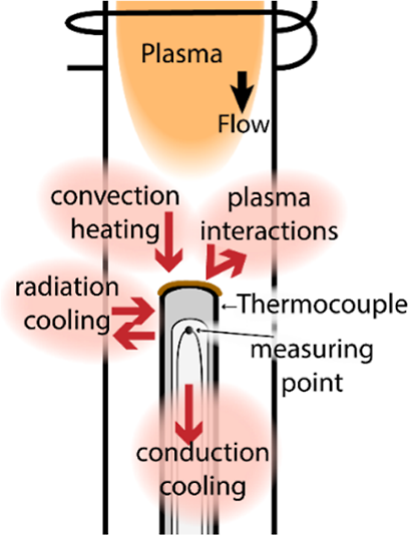
Heat fluxes on a thermocouple in a plasma flow reactor.

The heat fluxes affecting the probes are schematically shown in Figure 1, with the example of a single thermocouple (with a catalytic coating at the tip) in a flow reactor. Plasma interactions (including recombination), convection heat exchange between the gas and the probe, conduction cooling along the probe, and radiation heat exchange with the plasma and rector walls are expected to impact the measured temperature of the catalytic probe.

The measured temperature of a thermocouple over time, including the impact of the various heat fluxes, can be described according to
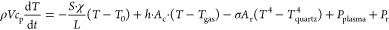
3where ρ*Vc*_p_ is the characteristics of the thermocouple (density, volume, and heat capacity respectively), *T* denotes the measured temperature of the thermocouple, and *t* denotes the time. The first term on the right-hand side of the eq 3 describes heat transfer due to thermal conductivity along the thermocouple, with *S* being the cross-sectional area of the thermocouple, χ its thermal conductivity, *L* the length over which there is a temperature gradient, and *T*_0_ the temperature at the end of this gradient (e.g., at the cold side of the thermocouple where it leaves the reactor). The second term denotes the convective heat transfer, describing the energy transferred at the interface between the gas and the thermocouples, with *h* being the heat transfer coefficient and *A*_*c*_ being the surface area of the thermocouple maintained at different temperatures from the flowing gas. The third term represents heat transfer through thermal radiation, with σ being the Stefan–Boltzmann constant and *A*_r_ being the surface area of the probe with a different temperature than the surrounding quartz glass tube, which has *T*_quartz_ as the temperature. *P*_plasma_ represents an accumulation of heating processes from the plasma on the thermocouple, while *P*_r_ specifies recombination heating on the catalyst.

Various ways of distinguishing the different heat fluxes are described in the literature, from purely analytical to mechanical. The analytical approach is based on the evaluations of cooling measurements for estimating heat fluxes. Here, single catalytic probes are used (fiber optic catalytic probes or thermocouples).^6,14,16,17,22^ The analysis is based on two assumptions taken at thermal equilibrium: (i) The only source of heating the catalytic probe comes from recombination and (ii) the heating of the probe is equal to the thermal losses *P*_c_ due to convection, conduction, or radiation; thus, *P*_r_ = *P*_c_.^6,18^ The combination of cooling processes *P*_c_ is simplified to
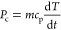
4with *mc*_p_ being the thermocouple’s characteristics (mass and heat capacity respectively of the part with thermal gradient).  is obtained by fitting the temperature over time in the initial cooling phase after the plasma is switched off. Then, *nv* becomes^18^
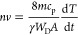
5

Disadvantages of this evaluation are the uncertainty of the length of the temperature gradient, translating to uncertainties in the value for *m* and the variation in cooling time scales of the different cooling processes giving varying results for  for different choices of cooling time in the fitting.

To avoid ambiguities of the fitting approaches by gaining more information on the heat fluxes, dual probe approaches have been suggested.^9,12,15^ A catalytic probe is paired with an identical reference probe without catalyst coating, measuring the temperature simultaneously. Carruth et al.^12^ proposed a dual probe design using two encased thermocouples. The reference side had a resistive heater next to the thermocouple inside the encasing, bringing the reference side to the same temperature as the catalyst side. With both sides at the same temperature, the heat losses are the same, and the information on their magnitude is contained in the power needed to heat the reference side. A disadvantage of this design is the rather large size of the probes. Qerimi et al.^9^ proposed a dual probe approach with two simple thermocouples. In their evaluation, they assumed the only relevant thermal loss process to be thermal conduction, leading to the evaluation function^9^
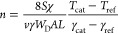
6with *S* being the cross-section of the thermocouples, χ the thermal conductivity, *T*_cat_ and *T*_ref_ the measured temperatures of the catalytic and the reference probe, respectively. γ_cat_ and γ_ref_ are the corresponding recombination coefficients. *L* denotes the thermal gradient length of the probe, showing that this approach still has the disadvantage of having to know the length of the temperature gradient.

In this paper, we propose a new probe design, reducing uncertainties in the current evaluations such as the length of the thermal gradient *L*, probe properties, and the impact of heat radiation. Measurements shown here use the example of Nitrogen plasma, forming N atoms as radicals. However, the probe design is transferable to other systems. We present a heat flux analysis of the system, comparing measurements of the probe with infrared imaging of the reactor walls. The probe is based on three thermocouples, adding a heat sink monitored by a thermocouple to a dual thermocouple setup. This facilitates good control of *L* and helps us gain more information on the various heat fluxes. This setup facilitates the estimation of the radiation heat flow without knowing the reactor wall temperature. Furthermore, we mount the probe on an adjustable feedthrough making it possible to map the density throughout the reactor, gaining insights into radical recombination kinetics at varying pressure and flow rate settings.

## Experiments

**Figure 2:**
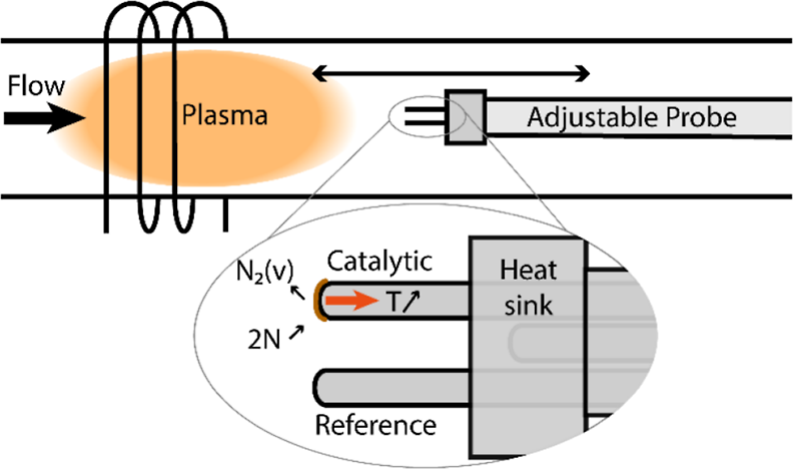
Schematic of the setup, with zoom-in on the (length) adjustable probe consisting of three thermocouples in contact with a heat sink.

The experiments were performed in an RF inductively coupled flow reactor (as described in refs (23,24)). A simplified schematic of the reactor and probe is depicted in Figure 2. An RF generator of T&C Power Conversion Inc., type AG 0313 was combined with a matching box of type MIT-600-3 as power input to the coil. Mass flows of 30 to 100 sccm of N_2_ were applied to the reactor using Brooks Instruments type GF040CXXC mass flow controllers. The quartz tube had a length of 75 cm and an inner diameter of 32 mm. A vacuum pump at the gas outlet kept the system at a low pressure. Experiments were done at 3, 5, and 7 mbar.

For the probe, three K-type thermocouples of 1 mm diameter and 1.5 m length in a stainless steel housing (1.4841) were mounted through an adjustable feedthrough with a 50 cm long stainless steel tube that could move parallel to the gas flow direction. At the tip of the stainless steel tube, the heat sink is mounted, a cup-shaped stainless steel piece that could be slid on and fixed with a screw, having good thermal contact with the tube. The heat sink has 3 holes. 2 of them penetrated the sink. Through these, the thermocouples were fed (the holes were only slightly larger than the thermocouples, ensuring thermal contact between the thermocouples and the heat sink). The third hole in the heat sink penetrates it only partially. Here, the third thermocouple is pushed in to measure the sink temperature.

The thermocouples were identical except for a 200 nm thick copper (Cu) coating on one of them, the catalytic thermocouple. The Cu was applied only to the tip by a magnetron sputter coating. For this, the tip was fed through a piece of metal, behind which the rest of the thermocouple was rolled up. Masking tape was applied around the part of the thermocouples sticking out of the metal disk so that only the tip was subjected to the sputtering.

For mapping the N density throughout the reactor, plasma was ignited with the probe in the position farthest away from the plasma glow. Knowing the half-time for a temperature change of the reactor is about 10 min, and temperature stabilization was allowed for 30–40 min before starting the probe measurement. For the density mapping, the probe was moved in steps toward the plasma glow. The measured time for each of these steps was long enough to ensure that the catalytic and reference thermocouples exhibited the same temperature gradient. This condition is necessary for the validity of the evaluation (eq 11), as it relies on both gradients being identical. The last step of each measurement was a repeat measurement at the initial distance (farthest from the plasma). This is an activation check: if the temperature difference between the reference and catalytic thermocouple is the same both times, then the probe did not degrade during the measurement. At each step, a picture was taken with a fixed webcam from the above. This allowed for accurate measurement of the probe distance to the plasma.

For the heat flux analysis, measurements of the surface temperature of the quartz tube were performed by using a FLIR IR camera. The side of the quartz tube facing the camera was sprayed with a thermographic paint (LabIR) with a high and well-defined emissivity coefficient. Temperature readings were taken from the part of the quartz tube image where the glass faces the camera perpendicularly.

## Results and Discussion

To study the heat fluxes affecting the temperature measurement, we conducted several tests in a dual probe setup (without a heat sink). We measured the heating and cooling of the thermocouples in the reactor with plasma on and off while simultaneously measuring the temperature of the quartz tube on the outside with an IR camera.

**Figure 3:**
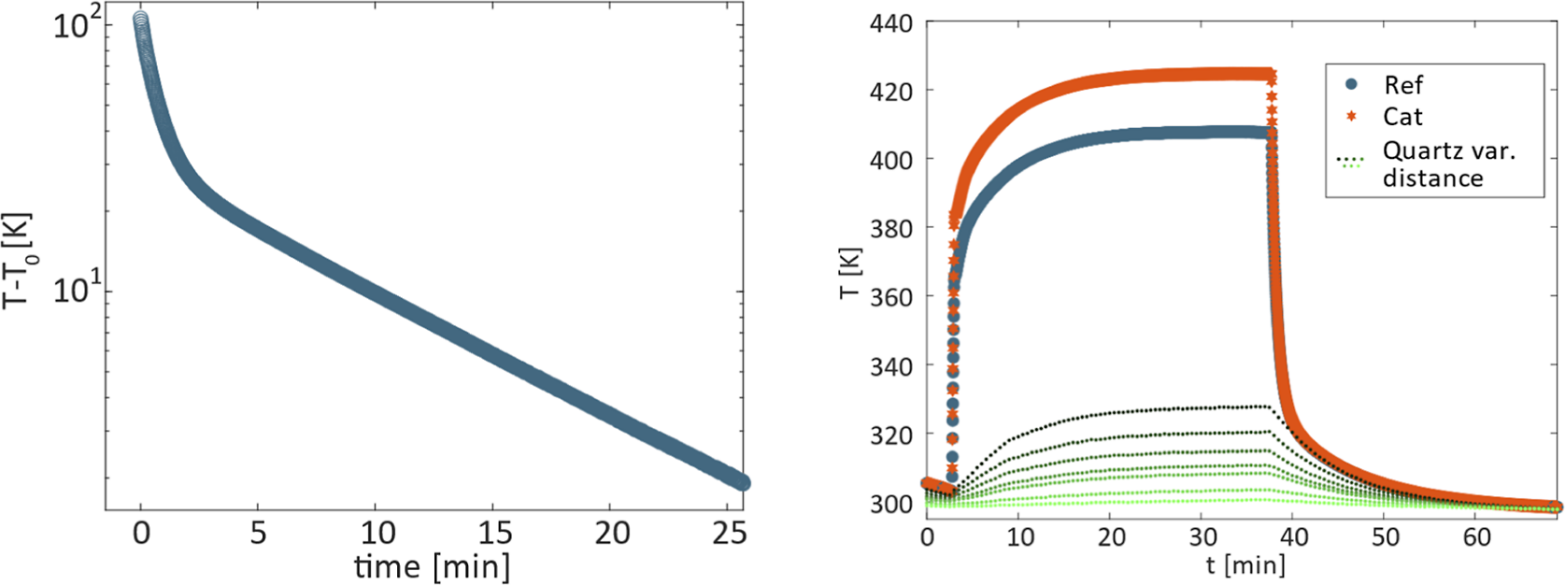
Left: Temperature over time for cooling after switching off the plasma *T*_0_ is room temperature; right: temperature evolution with plasma on and off for thermocouples at 7.9 cm from the coil and quartz surface temperature at varying (var.) distances (5.5–12 cm) from the coil.

The logarithmic plot of measured temperature change over time (Figure 3, left) reveals two distinct time constants which can be fitted to about 1 and 10 min (fitting ). Thermal imaging of the quartz tube during cooling showed a cooling time constant of 10 min, thus matching the second time constant in the thermocouple measurement. This is confirmed by plotting simultaneous measurements of quartz temperature and thermocouples (Figure 3, right). It is obvious that the cooling of the thermocouples is governed by the cooling of the quartz 2 –3 min after the plasma is switched off. This suggests a significant impact of radiation cooling on the measured thermocouple temperature.

**Figure 4:**
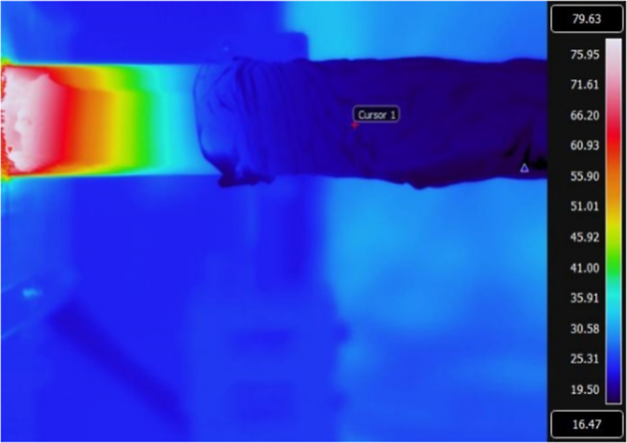
IR image of cooling the quartz tube around the thermocouple while plasma is on.

**Figure 5:**
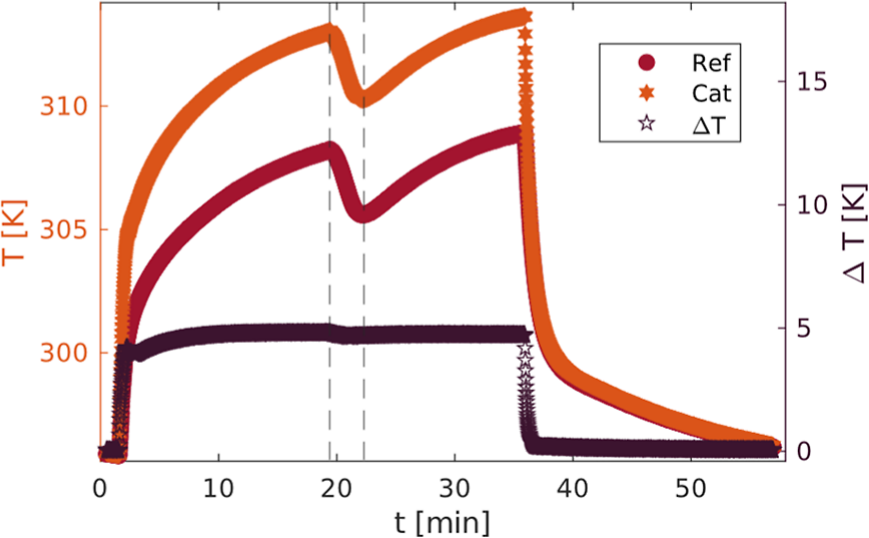
Temperature of the thermocouples with plasma on (from minute 1–36) and cooling of the quartz around minute 20.

Further tests of the impact of radiation on the measured temperature were done by cooling the quartz tube with a wet cloth, while the plasma was switched on. At the experimental conditions of 5 mbar and 100 sccm mass flow, the gas flow is laminar so that we expect this effect to be dominated by the radiation cooling of the thermocouples. An IR image of the experiment is depicted in Figure 4. Figure 5 shows the thermocouple temperature log during this test. Around minute 20, the quartz tube was cooled. It can be seen that the cooling of the quartz tube has a large effect on the measured temperature of each of the thermocouples individually, but only negligible effect on the Δ*T* between the thermocouples (with Δ(Δ*T*) < 1 K). This shows that a dual thermocouple probe is useful in distinguishing heat flows. Furthermore, we see that single probe designs are strongly impacted by radiation, and thus this must be included in the heat flux analysis. Repeated testing under vacuum conditions (no flow, 10^–2^ mbar pressure) with external heating and cooling showed the same large effect of quartz temperature on thermocouple temperature, excluding a possible impact of convection on the previous result.

Convective heat transfer causes heating of the probe from the interaction of the gas with the probe surface. This form of heat transfer results from a combination of diffusive heat transfer (conduction) and bulk motion of the gas (advection).^25^ Newton’s law of cooling is commonly used to describe convection and can also be found in the second term of eq 3 (temperature of a thermocouple over time). As the thermocouples are heated by the gas, the difference in temperature between the thermocouple surface and the gas temperature decreases. At equilibrium conditions, the difference between gas and thermocouple temperatures is small; thus, this term can be neglected.

Conduction heat transfer along the thermocouple depends on the temperature gradient (temperature difference and length of the conductive material, see eq 3). In the evaluation techniques described in the literature, this is represented by mass *m* in eq 5 and length *L* in eq 6. In our system, the thermocouples have a length of about 60 cm between the plasma and exit of the reactor. As the temperature gradient must be expected to be shorter than this, knowledge of *m* or *L* requires more information. Fitting the cooling according to eq 3 (without convection term) to obtain an estimation for this value did not give unique results as the conduction and convection terms showed to be interdependent. To obtain further information about the system, we added a heat sink with a third thermocouple to the dual thermocouple setup (see Figure 2) so that the length of the thermal gradient can be controlled and the temperature difference can be monitored throughout the experiment.

To account for the temperature gradient in the radiation term, we assume a linear gradient so that the temperature *T*_TC_ over the length is described as
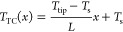
7with *T*_tip_ being the temperature measured by the thermocouple, *T*_s_ the temperature of the heat sink, and *L* the length of the thermocouple sticking out of the heat sink (and thus of the thermal gradient). For the radiation heat transfer *P*_rad_, the temperature of the surface area needs to be taken into account. For this, we integrate over the curved surface area
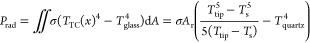
8Here, σ is the Stefan–Boltzmann constant and *T*_glass_ is the temperature of the reactor wall facing the thermocouples. We omit the surface area of the tip, as it is only a small part of the total area and does not face the quartz directly. Note that the tip faces the plasma, which in this application is cold, so significant heating at this surface is not anticipated. This can be different for hot plasmas.

The temperature over time for catalytic and reference thermocouples then becomes:

1. reference thermocouple
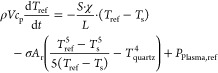
9

2. catalytic thermocouple
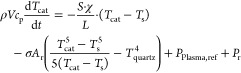
10Here, *T*_ref_ and *T*_cat_ are the temperatures measured by the reference and catalytic thermocouple, respectively. *P*_r_ is the heating power from recombination on the catalyst surface, and *P*_Plasma,ref_ is the heating power from plasma interactions on the stainless steel surface of both thermocouples. As only a small fraction of the catalytic thermocouple (the tip) is coated with a catalyst, we assume that this term is the same for both thermocouples. For the evaluation of *P*_r_, the temperature measurement must continue until a stationary thermal equilibrium is reached  or both catalytic and reference thermocouple change in temperature at the same rate (), which happens much quicker in the experiment. With this condition met, subtracting eqs 9 and 10 gives the heating power from recombination on the catalytic surface as
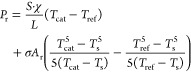
11

Note that the quartz temperature is not in the equation. This means that almost all variables needed for the calculation are known. Only the thermal conductivity χ of the thermocouple needs to be estimated. The thermocouples used in this experiment are filled with MgO. SEM analysis showed that they had a stainless steel wall thickness of 125 μm. The thermal conductivity of steel is 15 W/(Km).^26^ The thermal conductivity of MgO is temperature dependent (30 W/(Km) at 400 K and 60 W/(Km) at 400 K).^27^ To increase the accuracy of the evaluation, we estimate the χ_Mg_ of MgO for each measurement using the mean temperature between the thermocouple and the heat sink (assuming a linear relationship of χ_Mg_ with temperature). The heat conductivity of the thermocouple χ is then calculated by using the volume ratio of MgO and stainless steel. Assuming an error of ±2 W/(Km) for the thermal conductivity of stainless steel and ±8 W/(Km) for χ_Mg_, the resulting error in χ for typical temperature values during our measurements is about 7%.

During measurements, we observed the radiation term to be less than 10% of the conduction cooling for an *L* of 10 mm. We chose a small *L*, as for a larger *L*, the radiation term is expected to play a larger role. The lifetime of the probe was about 20 h at low temperatures. When using an oven to heat the reactor to 600 °C, the Cu catalyst surface was poisoned already at the beginning of the experiment.

An advantage of using catalytic and reference thermocouples is faster equilibration times. Equilibration to the same heating rate of the catalyst and reference thermocouple was for most conditions obtained after 2 min, while equilibration to a stable temperature for either of the thermocouples lasted >10 min.

The largest contributor to uncertainty in radical density (see eq 2) is generally stated as the recombination coefficient γ.^22^ This is a systematic uncertainty and is known to depend on factors, such as surface morphology, possible oxide contamination, pressure, and temperature. Therefore, it should ideally be measured independently.^18^ The effective recombination coefficient for a probe consisting of reference and catalytic side is the difference between the recombination coefficients of the two: γ_eff_ = γ_cat_ – γ_ref_. In this study, we unfortunately have no means to measure recombination coefficients and must rely on literature values for N recombination on copper γ_cat_ = 0.18^15^ and for stainless steel γ_ref_ = 0.005.^28^ However, we tested the sputtered copper coating to confirm a pure coating, without impurities (which are expected to strongly alter the recombination coefficient). For best results, γ_ref_ should be minimized (with regard to the radical measured) by coating the surface of both the thermocouples and the heat sink with a material of low recombination probability (e.g., MgO or Al_2_O_3_) before applying the catalyst to the tip of the catalytic thermocouple. This approach increases the measured temperature difference and reduces uncertainties originating from the thermocouple measurement. The recombination coefficient of N on stainless steel is low; therefore, this step is not necessary for our measurements. Another possible source of error is the dissociation energy W_D_, which might be overestimated, as the recombined molecules often leave the surface vibrationally excited.^29,30^

The atom density of nitrogen is calculated by combining eqs 2 and 11
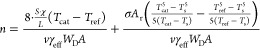
12

**Figure 6:**
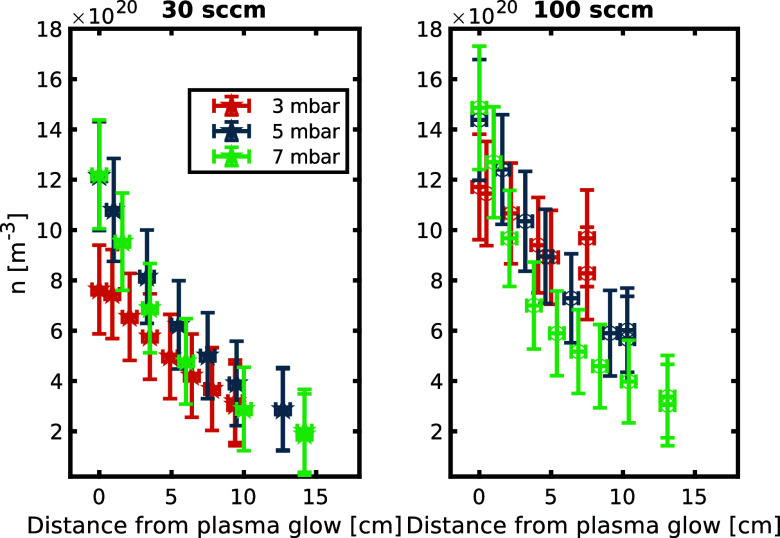
Radical density of N over distance from plasma for various conditions.

The map of N density over the distance from the plasma glow is plotted in Figure 6. The probe can resolve a decay in density over the reactor. It shows that a larger flow rate carries the species further and that a larger pressure leads to a shorter volume with active species (note that the gas flow speed reduces with increased pressure when mass flow and temperature remain constant). Measurement uncertainty taken into account in the plot originates from thermal conductivity χ (see above), length *L* (±1 mm), as well as the measured temperatures *T*_ref_, *T*_cat_, *T*_s_ (±0.4 K). Here, the thermal conductivity has the largest effect on the uncertainty. Errors in γW_D_ are difficult to estimate as they strongly depend on surface cleanliness and morphology. Assuming an error of 20% for γW_D_ would increase the error bars (from now approximately 15%) to 35%.

An advantage of space-resolved measurements is the option to evaluate reaction kinetics. It also opens the option to check if the measured signal is caused by radical recombination, rather than other interactions. The pathway for recombination of N radicals in the gas phase is via three-body recombination (N + N + M → N_2_ + M).^28,31^ The rate equation over distance is
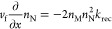
13with *v*_f_ being the gas flow velocity, *x* the distance to the plasma glow, *k*_rec_ the recombination rate, and *n*_M_ the concentration of the third particle (in this case N_2_). Solving for 1/*n* yields
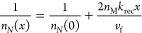
14

**Figure 7:**
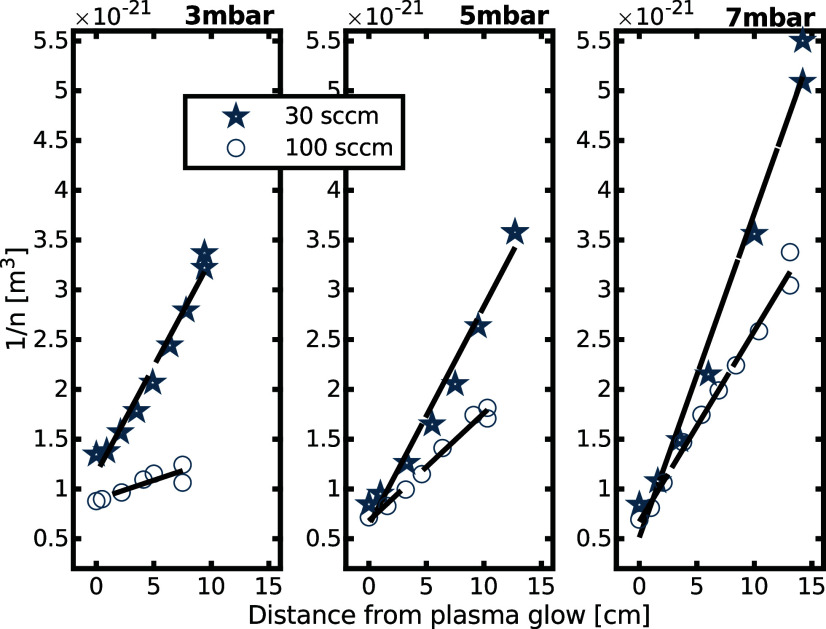
Fitting of radical density shows that the measured data are in line with the theory on three-body recombination.

A fit of the measured data 1/*n* over distance is shown in Figure 7. In all measured conditions (3, 5, and 7 mbar and flow rates 30 and 100 sccm), the data match the model of three-body recombination well, giving a recombination rate of *k*_rec_ = (2.0 ± 0.9)·10^–44^ m^6^/s, which is in line with literature values.^31^ This shows that the probe measures radicals and does not significantly disturb the processes in the afterglow.

## Conclusions

The analysis of the impact of the system’s heat fluxes on the measured temperature of catalytic probes revealed that the radiation significantly influences the temperature readings in a single thermocouple setup. For a dual thermocouple setup (adding a reference thermocouple), evaluating the temperature difference between the thermocouples showed to reduce the impact of radiation on the measurement but did not solve ambiguities in determining the probe characteristics and conductive heat loss. The addition of a heat sink with an integrated heat sensor can reduce these ambiguities by (i) enabling control over the length of the thermal gradient and (ii) giving information on the temperature difference over this gradient. Additionally, it enables the estimation of the impact of radiation heat flux without the necessity to know the reactor wall temperature. This three-thermocouple setup facilitates rapid mapping of the reactor (3–10 min per step) when mounted on an adjustable feedthrough. Measurements with spatial resolution of the N density over the reactor length allow for a kinetic study of N recombination. This demonstrated that the recombination reactions occur in the gas phase through three-body recombination, confirming that the probe does not influence the afterglow in the reactor during the measurements.
